# Pathologic response evaluation of localized or locally advanced esophageal carcinoma to induction chemotherapy followed by preoperative concurrent chemotherapy and hypofractionated radiotherapy: a clinical trial

**DOI:** 10.3389/fonc.2024.1439730

**Published:** 2024-08-19

**Authors:** Ali Emadi Torghabeh, Seyed Amir Aledavood, Ehsan Soltani, Mahsa Akbari Oryani, Saeed Akhlaghi, Sare Hosseini, Azar Fani Pakdel, Ali Taghizadeh Kermani, Kazem Anvari, Soodabeh Shahidsales, Shahrzad Bahadorian, Shervin Mashreghi Moghaddam

**Affiliations:** ^1^ Cancer Research Center, Mashhad University of Medical Sciences, Mashhad, Iran; ^2^ Surgical Oncology Research Center, Mashhad University of Medical Sciences, Mashhad, Iran; ^3^ Department of Pathology, Faculty of Medicine, Mashhad University of Medical Sciences, Mashhad, Iran; ^4^ Department of Biostatistics, School of Health, Mashhad University of Medical Sciences, Mashhad, Iran

**Keywords:** concurrent chemoradiotherapy, esophageal cancer, hypofractionated radiotherapy, induction chemotherapy, pathologic response

## Abstract

**Objective:**

Esophageal cancer is a therapeutic challenge in most healthcare systems. Most patients present with locally advanced disease at diagnosis. Concurrent chemoradiotherapy (CRT) is the standard treatment for locally advanced esophageal carcinoma. Since achieving a complete pathological response in postoperative specimens following neoadjuvant therapy is associated with improved patient survival, this study was designed to evaluate the pathologic response of localized or locally advanced esophageal carcinoma to induction chemotherapy followed by preoperative concurrent chemotherapy and hypofractionated radiotherapy (HFR).

**Methods:**

This single-arm clinical trial (IRCT20210623051676N1) evaluated patients with squamous cell carcinoma or adenocarcinoma of the esophagus, stage cT2-T4a N0 M0 or cT1-T4a N+ M0. Patients received 3-5 cycles of weekly induction chemotherapy with the paclitaxel (50 mg/m2) and carboplatin (AUC=2) regimen, followed by weekly concurrent CRT with the same chemotherapy regimen. The radiation dose was 40 Gy, delivered over 16 fractions, 5 days per week (2.5 Gray/fraction). Patients underwent surgery 4-6 weeks after completion of CRT. The surgical specimens were evaluated for pathological response. A p-value of < 0.05 was considered significant in all analyses.

**Results:**

Out of 54 patients enrolled in this study, 45 completed the neoadjuvant protocol. Of these 45 patients, 32 underwent surgery and were finally analyzed. The mean age of the patients was 59.9 ± 8.6 years (range, 37-75 years). The location of the tumor was in the mid-thoracic esophagus in most patients (21, 65.6%) and the most common histological type was SCC (29, 90.6%). The median number of induction and concurrent chemotherapy cycles was 5 (4.8 ± 1.3 course, range, 1-10) and 3 (2.6 ± 0.8 course, range, 0-4), respectively. Among 45 patients who completed the neoadjuvant protocol, the most common toxicities were grade 3 neutropenia (15.6%), acute renal failure (4.4%), and odynophagia (37.8%). Nearly two-thirds of the patients experienced complete or near-complete responses (71.9%, 23 patients). Partial response was reported in 6 patients (18.8%) and poor response in 3 patients (9.4%).

**Conclusion:**

Preoperative induction chemotherapy followed by HFR with concurrent chemotherapy has low toxicity and side effects, good tolerance, and significant efficacy in the treatment of patients with esophageal cancer.

**Clinical trial registration:**

https://irct.behdasht.gov.ir/trial/59930, identifier NCT05745545.

## Introduction

Esophageal cancer is a prevalent and aggressive malignancy, encompassing two main histological subtypes: Esophageal Squamous Cell Carcinoma (ESCC) and Esophageal Adenocarcinoma (EAC). ESCC and EAC exhibit distinct epidemiological and clinical characteristics. ESCC is particularly prevalent in the northern and northeastern regions of Iran (1, 2). Treatment options for this disease have evolved, with a growing focus on neoadjuvant chemoradiotherapy (CRT). CRT combines chemotherapy and radiotherapy (RT) before surgery, offering several advantages, and several studies have demonstrated that neoadjuvant CRT leads to superior pathological complete response, regional control, and overall survival compared to esophagectomy alone (3–5).

Several studies have shown the prognostic significance of a complete pathologic response after initial treatment for esophageal cancer patients on survival outcomes (6–13).

Conventional fractionated radiotherapy (CFR) has been the standard neoadjuvant treatment. However, its prolonged duration and associated costs raise concerns. Hypofractionated radiotherapy (HFR) offers an alternative approach, delivering higher doses than 2 Gy per fraction with fewer overall fractions. HFR has proven effective in treating other cancers like prostate, breast, and locally advanced head and neck cancers, and its potential in esophageal cancer is gaining support (14–17).

Several studies have demonstrated the safety and efficacy of HFR versus CFR in esophageal cancer patients in terms of survival, pathologic response, reduced treatment time, and decreased healthcare costs (18–21).

The effectiveness of adding induction chemotherapy before chemoradiation for resectable esophageal cancer is still unclear. While some studies show potential benefits (22), others do not (23). However, using induction chemotherapy reduces dysphagia and improves radiotherapy treatment tolerance, especially when there are delays or limitations in accessing radiation therapy.

Due to the relatively high prevalence of esophageal carcinoma in the Khorasan region and the scarcity of RT resources, researchers hope to be able to increase the effectiveness of pre-operative treatments by using an induction chemotherapy program and a shorter course of RT, while also reducing costs. Therefore, this study was conducted to evaluate the pathological response of localized or locally advanced esophageal carcinoma to induction chemotherapy and concurrent chemotherapy with HFR before surgery.

## Materials and methods

This clinical trial enrolled patients with esophageal cancer who had undergone endoscopic biopsy confirming the diagnosis of ESCC or EAC and had presented to the Omid, Imam Reza, and Hashemi Nejad hospitals in Mashhad between 2021 and 2023. Patients underwent staging evaluation with barium swallow and chest and abdominal CT scans to assess local and distant metastasis. Patients suspected of having T1 or T4 primary tumors based on these evaluations also underwent endoscopic ultrasound. Patients with cT2-T4a N0 M0 or cT1-T4a N+ M0 primary tumors and patients with grade III dysphagia or higher were included in the study (based on the study by Fang and colleagues (24), grade III dysphagia or higher in patients with esophageal cancer predicts T3 and higher lesions with 100% specificity). Routine laboratory tests, including complete blood count, liver and kidney function tests, and serum albumin levels, were also evaluated for these patients. The inclusion criteria encompassed the following aspects: (a) patients with carcinoma in the thoracic or abdominal esophagus, (b) patients with performance status ≥70% by Karnofsky criteria or ≤1 by ECOG criteria, (c) patients with grade III dysphagia or higher or stage cT2-T4a N0 M0 or cT1-T4a N+ M0 disease based on staging findings, and (d) patients with consent to complete the entire treatment plan, including surgery. Exclusion criteria comprised: (a) patients with metastatic disease, (b) patients with cervical esophageal carcinoma, (c) patients with tumors invading the heart, trachea, bronchus, or aorta, (d) patients who refuse to undergo surgery, and (e) patients with previous history of cancer or other comorbidities that would prevent completion of the treatment plan.

All participants provided informed consent. The study protocol was approved by the Ethical Committee of Mashhad University of Medical Sciences (IR.MUMS.MEDICAL.REC.1400.482). This clinical trial was registered in the Iranian Clinical Trials Registry (IRCT20210623051676N1).

Participants underwent three to five cycles of weekly induction chemotherapy with a combination regimen of paclitaxel (50 mg/m2) and carboplatin (AUC=2), followed by weekly concurrent CRT with the same regimen for three cycles and RT at a dose of 40 Gy with 2.5 Gy fractions over 16 sessions, five sessions per week. RT was delivered using the three-dimensional conformal radiotherapy (3DCRT) technique with a linear accelerator. The treatment volumes consisted of Gross Tumor Volume (GTV) which included the volume of the primary esophageal tumor and involved regional lymph nodes based on findings from endoscopy, barium swallow, CT scan, and endoscopic ultrasound (if performed); Clinical Target Volume (CTV) which included areas with potential tumor involvement, obtained by adding a 1 cm radial margin and a 3 cm longitudinal margin to the GTV on each side, while not exceeding anatomical boundaries; and Planning Target Volume (PTV) which was obtained by adding a 0.5 to 1 cm margin to the CTV in all directions to account for uncertainties.

Patients were visited by their treating physician once a week to assess and record any potential treatment-related side effects. Laboratory tests, including complete blood count and kidney function tests, were requested for the patients, and the results were recorded in their medical records. During RT, in case of severe side effects and the need to halt the treatment, a maximum one-week treatment break was acceptable, and the patient remained in the study. However, if the delay was more than one week and the patient did not agree to surgery or could not tolerate surgery, the patient was withdrawn from the study. After 4-6 weeks of completing neoadjuvant treatment, patients underwent esophagectomy either transhiatally or transthoracically, and the surgical specimens were evaluated for pathological response.

**Figure 1:**
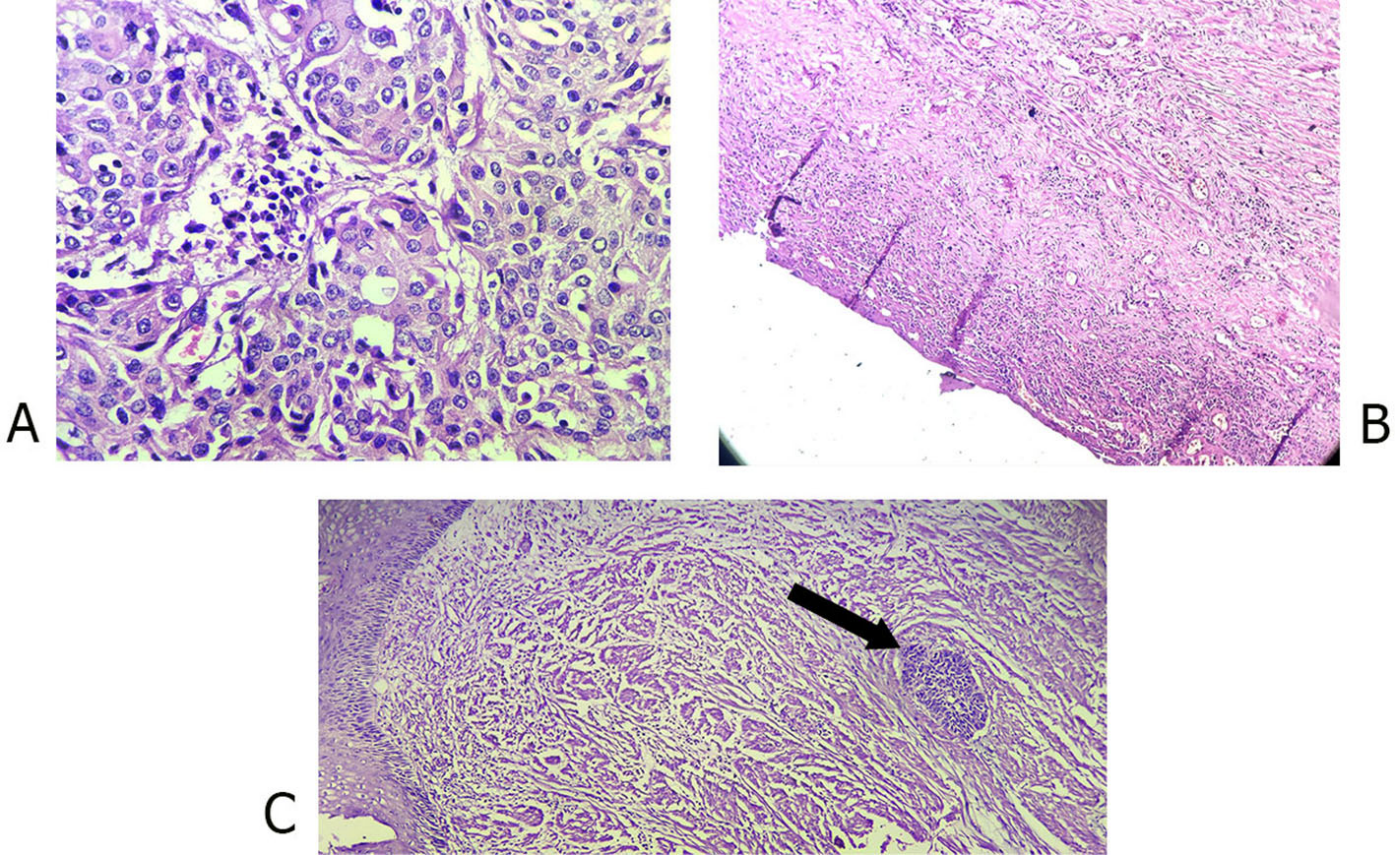
Photomicrographs of **(A)** Poorly differentiated esophageal squamous cell carcinoma, pretreatment (40x); **(B)** Complete pathologic response after treatment (10x); **(C)** Near complete response after treatment (arrow head showing small group of cancer cells).

Tumor response to preoperative treatment was pathologically evaluated using the College of American Pathologists (CAP) criteria (25) (**Table 1**) (**Figure 1**) Post-surgical complications were monitored and recorded for up to one month (**Table 2**).

**Table 1:** College of American Pathologists (CAP) criteria, tumor regression score in esophageal cancer.

Description	Tumor Regression Score
No viable cancer cells (complete response)	0
Single cells or rare small groups of cancer cells (near complete response)	1
Residual cancer with evident tumor regression, but more than single cells or rare small groups of cancer cells (partial response)	2
Extensive residual cancer with no evident tumor regression (poor or no response)	3

**Table 2:** Characteristics of neoadjuvant therapy-related adverse events and acute surgical complications in the studied patients.

Variable	Patients who have undergone surgery(n=32)	Non-operated patients (n=13)	Total (n=45)
Hematologic			
Neutropenic fever	0 (0%)	0 (0%)	0 (0%)
Grade 3 thrombocytopenia	0 (0%)	0 (0%)	0 (0%)
Grade 3 neutropenia	4 (12.5%)	3 (23.1%)	7 (15.6%)
Grade 3 anemia	0 (0%)	0 (0%)	0 (0%)
Acute kidney injury	1 (3.1%)	1 (7.7%)	2 (4.4%)
Oral mucositis	0 (0%)	0 (0%)	0 (0%)
Odynophagia			
No odynophagia	10 (31.3%)	7 (53.8%)	17 (37.8%)
Grade 1 odynophagia	8 (25%)	3 (23.1%)	11 (24.4%)
Grade 2 odynophagia	11 (34.4%)	3 (23.1%)	14 (31.1%)
Grade 3 odynophagia	3 (9.4%)	0 (0%)	3 (6.7%)
Acute Surgical Complications			
No complications	25 (78.1%)	–	–
Pleural effusion	2 (6.3%)	–	–
Sepsis	3 (9.4%)	–	–
Anastomotic leak	1 (3.1%)	–	–
Hydropneumothorax	1 (3.1%)	–	–

" - " means this is not applicable for those patients.

The sample size was estimated based on the AleDavood et al. study’s findings (26). Considering a 52% combined complete and relative success rate, 5% type I error, and 15% precision, the estimated sample size was 43 patients. The collected data were analyzed with SPSS software version 21. Descriptive statistics were reported for continuous variables with normal distribution using mean ± standard deviation (SD), for continuous variables with non-normal distribution using median (interquartile range, IQR), and for categorical variables using frequency (N%) counts, normality of variables investigated by Shapiro Wilk. To compare categorical variables between the study groups, the chi-square test (or Fisher’s exact test) was used. The Mann-Whitney U test was used to compare numerical variables between two groups.

In all tests, p < 0.05 was considered statistically significant.

## Results

**Figure 2:**
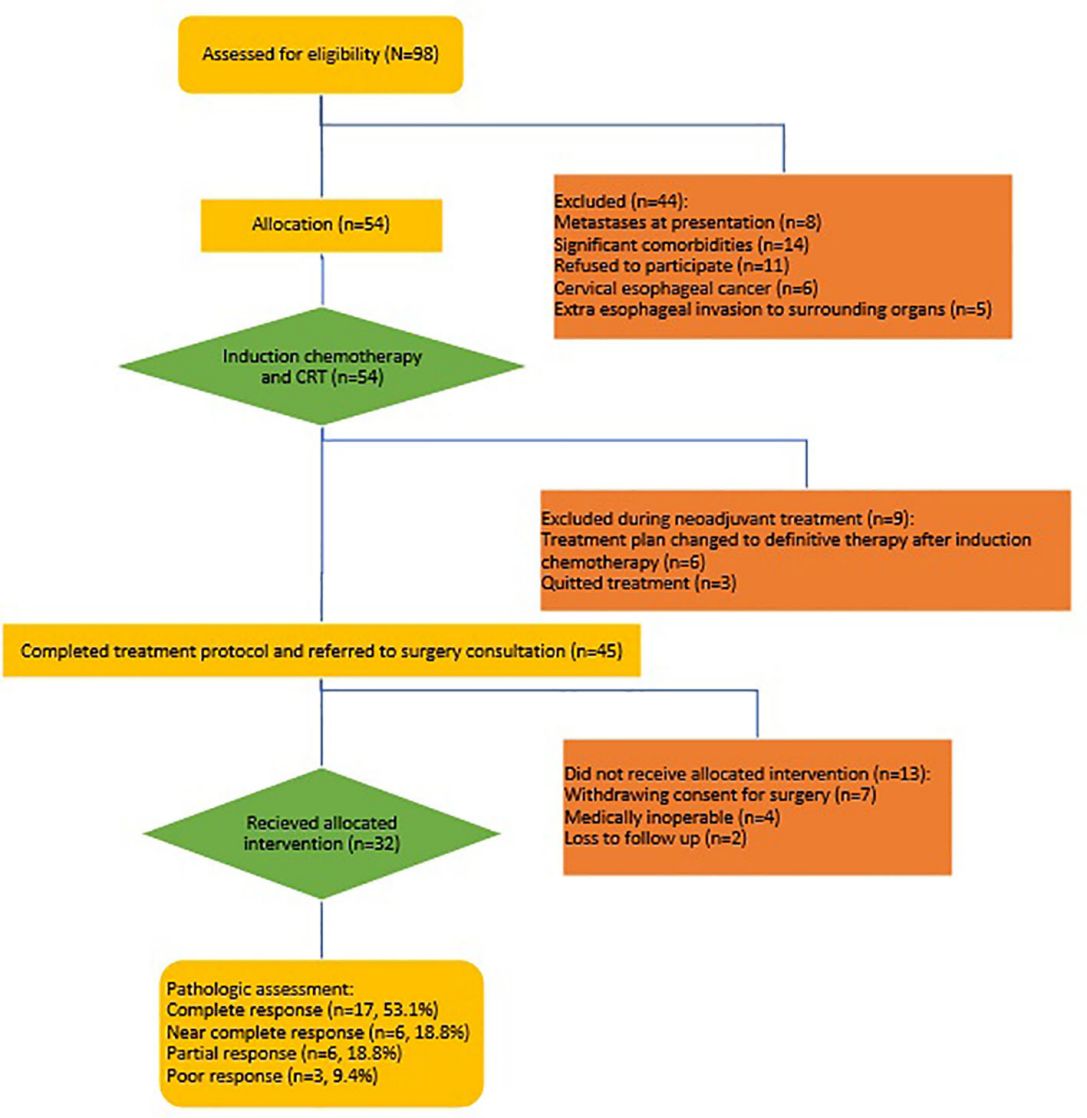
CONSORT diagram of patient flow in the study. CRT, chemoradiotherapy.

Between 2021 and 2023, 98 patients with esophageal cancer who were referred to the Omid, Imam Reza, and Shahid Hashemi Nejad hospitals in Mashhad were evaluated for eligibility criteria. Eight patients were excluded due to metastasis at diagnosis, 11 patients due to refusal to participate in the study, 6 patients due to a malignant lesion in the cervical esophagus, 5 patients due to evidence of invasion of the heart, trachea, bronchus, or aorta on preoperative imaging, and 14 patients due to uncontrolled underlying medical conditions including diabetes, hypertension, chronic kidney disease, and chronic liver disease were ineligible for the study. A total of 54 patients who met the inclusion criteria were enrolled in the study to receive the neoadjuvant treatment protocol of induction chemotherapy and concurrent hypofractionated CRT. Nine patients were excluded from the protocol during treatment. Six patients who initially responded to induction chemotherapy subsequently withdrew consent for surgery after radiotherapy due to their symptom resolution. These patients were then switched to a definitive chemoradiotherapy plan. Three patients discontinued treatment between induction chemotherapy and chemoradiotherapy. Among the 45 patients who completed the neoadjuvant treatment protocol, 13 were excluded from further analysis. Following CRT, two patients were lost to follow-up, four were medically unfit for surgery, and seven withdrew their initial consent for surgery due to symptom resolution and their concerns about surgery-related mortality and morbidity. 32 patients underwent surgery and were included in the final analysis (**Figure 2**).

The mean age of the patients was 59.9 ± 8.6 years (range 37-75 years). **Table 3** presents detailed demographic data for patients who completed the entire protocol, including surgery.

**Table 3:** Demographic characteristics of the studied patients who completed the protocol including surgery.

Variables	Frequency	Percentage
Gender		
Male	17	53.1%
Female	15	46.9%
Performance Status		
1	30	93.8%
2	2	6.3%
Dysphagia Grade		
2	5	15.6%
3	17	53.1%
4	10	31.3%
Tumor Location		
Middle thoracic	21	65.6%
Lower thoracic/Esophagogastric junction	11	34.4%
Pathology		
Squamous cell carcinoma	29	90.6%
Adenocarcinoma	3	9.4%
Tumor Grade		
1	11	34.4%
2	16	50%
3	5	15.6%

For patients who completed the protocol including surgery, the median number of induction chemotherapy cycles was 5 (4.8 ± 1.3, range 1-10). The median number of concurrent chemotherapy cycles was 3 (2.6 ± 0.8, range 0-4). **Table 4** shows detailed information on induction and concurrent chemotherapy cycles. Both treatments were well tolerated in most patients, with a delay in induction chemotherapy observed in only 4 patients (12.5%). There were no delays in CRT. The median time interval between induction chemotherapy and the start of CRT was 20.5 days (interquartile range 9.25-37.25 days), and the median time interval between CRT and surgery was 63 days (interquartile range 46.5-75.5 days) (**Table 4**).

**Table 4:** Characteristics of neoadjuvant therapy for esophageal cancer in patients who completed the protocol including surgery.

Variable	Frequency	Percentage
Number of induction chemotherapy cycles		
1	1	3.1%
3	1	3.1%
4	8	25%
5	18	56.3%
6	2	6.3%
7	1	3.1%
10	1	3.1%
Number of concurrent CRT cycles		
0	1	3.1%
1	3	9.4%
2	5	15.6%
3	21	65.6%
4	2	6.3%
Induction chemotherapy delay		
No delay	28	87.5%
7 days or less	3	9.4%
8-14 days	1	3.1%
15 days or more	0	0%
CRT Delay		
No delay	32	100%
Delay	0	0%

CRT, chemoradiotherapy.

Among patients who completed the neoadjuvant protocol (45 patients) the most common hematologic toxicity was grade 3 neutropenia (7 patients, 15.6%). Neutropenic fever was not observed in any patient. Among non-hematologic toxicities, acute kidney injury was reported in two patients (4.4%) and grade 2 or higher odynophagia in 17 patients (37.8%). Among patients who undergone surgery (32 patients) acute surgical complications were reported in seven patients (21.9%), with the most common complications being sepsis (3 patients) and pleural effusion (2 patients). Surgical complications were associated with mortality in four cases (one due to anastomotic leak and three due to sepsis) (**Table 2**).

**Figure 3:**
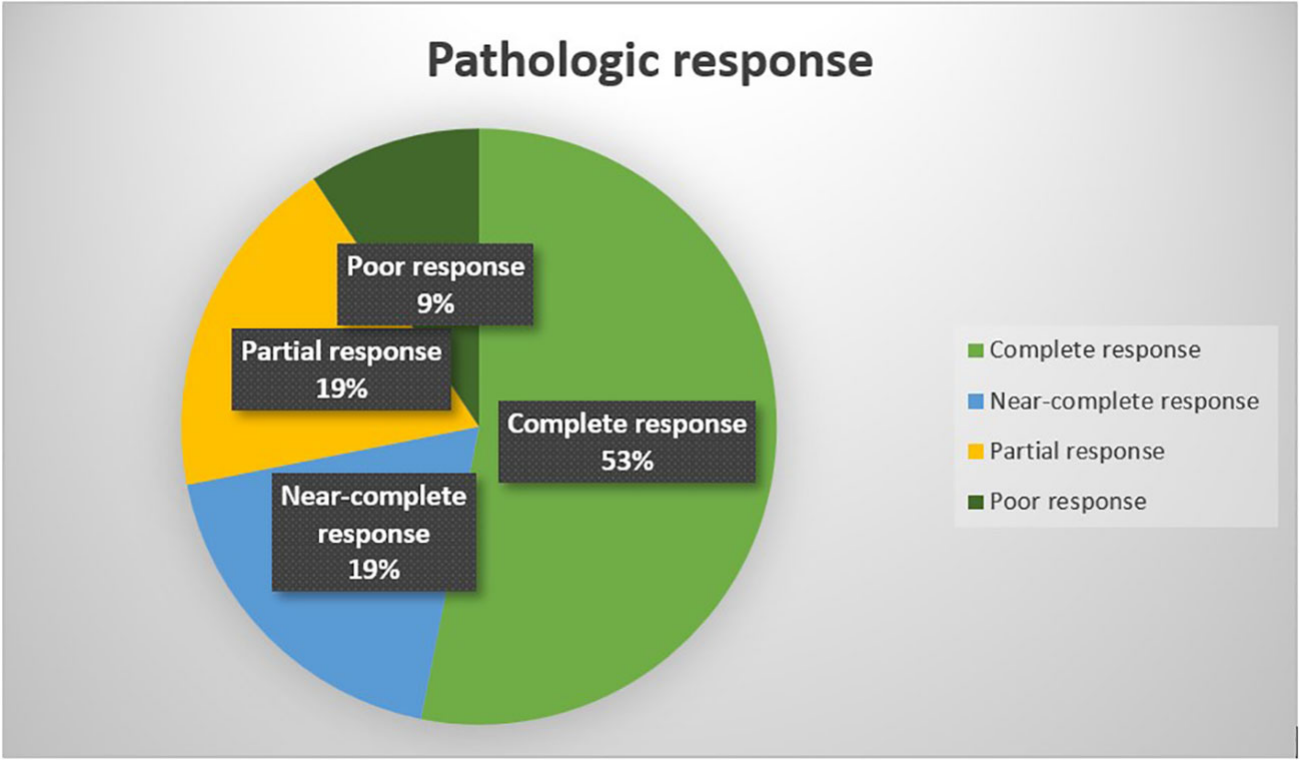
Pathologic response to neoadjuvant therapy.

Complete response was reported in 17 patients (53.1%), near-complete response in 6 patients (18.8%), partial response in 6 patients (18.8%), and poor response in 3 patients (9.4%) (**Figure 3**).

The frequency of complete and near complete responses in patients who received ≤4 cycles, 5 cycles, and ≥6 cycles of induction chemotherapy was 80% (8 patients), 66.7% (12 patients), and 75% (3 patients), respectively. The chi-square test revealed no statistically significant difference (p = 0.745). The frequency of complete and near complete responses in patients who received ≤2 cycles and ≥3 cycles of concurrent chemotherapy was 66.7% (6) and 73.9% (17), respectively. No statistically significant difference was found using the chi-square test (p = 0.682) (**Table 5**).

**Table 5:** Comparison of the association between pathologic response and number of concurrent and induction chemotherapy cycles.

Variable	Complete and Near Complete Response	Partial and Poor Response	P-Value
Induction chemotherapy cycles			
less than or equal to 4 cycles	8 (8%)	2 (2%)	0.745
5 cycles	12 (66.7%)	6 (33.3%)	
more than or equal to 6 cycles	3 (75%)	1 (25%)	
Concurrent chemotherapy cycles			
less than or equal to 2 cycles	6 (66.7%)	3 (33.3%)	0.682
more than or equal to 3 cycles	17 (73.9%)	6 (26.1%)	

**Table 6** shows no significant differences between complete/near complete responders and partial/poor responders in terms of time intervals between induction chemotherapy and CRT, or between neoadjuvant therapy completion and surgery (Mann-Whitney U test) (**Table 6**).

**Table 6:** Comparison of the association between pathologic response and the time interval between induction chemotherapy and initiation of CRT, as well as the time interval between completion of neoadjuvant therapy and surgery.

Variable	Complete and Near-Complete Response Median (days) (IQR)	Partial and Poor Response Median (days) (IQR)	p-value
Interval between Induction Chemotherapy and Initiation of CRT (days)	64 (45-77)	60 (54-76.5)	0.433
Interval between Completion of Neoadjuvant Therapy and Surgery (days)	20 (10-32)	28 (9-58.5)	0.869

SD, standard deviation; CRT, chemoradiotherapy.

## Discussion

In recent decades, neoadjuvant CRT has emerged as a promising treatment approach for esophageal cancer. It offers several advantages over surgery alone for esophageal cancer treatment. Firstly, adequate blood supply and oxygenation are crucial for effective chemotherapy and radiation sensitivity. Administering CRT before surgery preserves the tumor’s vascular network, thereby enhancing treatment efficacy. Secondly, CRT can shrink tumors, making surgical resection easier and reducing the likelihood of positive surgical margins. Thirdly, CRT-induced fibrosis and tumor cell destruction minimize the risk of tumor spread during surgery, consequently lowering the incidence of local recurrence. Finally, studies have demonstrated that neoadjuvant CRT leads to superior pathological complete response, regional control, and overall survival compared to esophagectomy alone (3–5).

Several studies have shown the prognostic significance of a complete pathologic response after initial treatment for esophageal cancer patients (6–12). Esophageal cancer patients, regardless of whether they have SCC or adenocarcinoma, who have residual tumor in their resection specimens following preoperative therapy tend to have poorer survival (7, 9, 13, 27). In a review of 235 cases where patients with esophageal or EGJ cancer underwent chemoradiation before surgery, post-treatment pathologic stage was the best predictor of survival (13).

CFR has become the standard approach for preoperative RT in esophageal cancer. However, concerns about prolonged treatment times and increased costs have led to the exploration of HFR as a potential alternative. HFR utilizes larger fraction sizes (greater than 2 Gy) compared to CFR while delivering a lower overall treatment dose. This approach offers several potential benefits: improved local tumor control through a higher effective biological dose to the tumor, and reduced normal tissue toxicity by considering the a/b ratio of healthy tissues (14, 28). Numerous studies have demonstrated the efficacy of HFR in various cancers, including lung (29, 30), prostate (31, 32), breast (17, 33), advanced head and neck (16, 34), and advanced unresectable esophageal cancer (15, 35). In particular, HFR has been extensively studied in the context of preoperative treatment for colorectal cancer. Research suggests that preoperative HFR (also known as short-course RT) is as effective as CFR (long-course RT) in terms of long-term survival (36–38).

The use of induction chemotherapy followed by chemoradiation for resectable esophageal cancer remains controversial. Ajani et al. found that patients with adenocarcinoma of the gastroesophageal junction (GEJ) who received induction chemotherapy before neoadjuvant CRT had a higher rate of pathologic complete response compared to those treated with preoperative CRT alone (22). Yoon et al. conducted a smaller, Phase II randomized trial comparing preoperative CRT with or without induction chemotherapy using S1 and oxaliplatin. Unlike the Ajani study, the majority (98%) of Yoon et al.’s patients had ESCC. This study did not demonstrate any improvement in pathologic complete response with the addition of induction chemotherapy (23). Additionally, initiating chemotherapy before CRT often results in symptom resolution, which can improve tolerance to CRT. CRT can cause esophageal inflammation and worsen swallowing difficulties, potentially necessitating interventions such as feeding tube placement. In settings with limited radiotherapy resources and long waitlists, induction chemotherapy can help alleviate symptoms.

The present study in patients with esophageal cancer who received induction chemotherapy followed by hypofractionated CRT revealed a pathologic complete and near-complete response rate of approximately 72%. Several studies have examined the efficacy of HFR-based neoadjuvant CRT for esophageal cancer.

Walsh et al. (19) conducted a randomized trial comparing surgery alone to a combination of HFR, chemotherapy, and surgery in patients with EAC. Patients in the intervention group received 40 Gy of RT over 15 fractions in 3 weeks followed by surgery. Their study found a pathologic complete response rate of 25% in the multimodality treatment group.

Lyu et al. (18) conducted a randomized trial in China comparing CFR and HFR-based neoadjuvant CRT for 110 patients with ESCC. Pathologic complete response rates were 78.6% and 83.8% in the CFR and HFR groups, respectively, with no statistically significant difference between the groups.

More recently, in 2024, Jiang et al. (39) investigated the efficacy of HFR-based neoadjuvant CRT with a dose of 30 Gy in 12 fractions combined with paclitaxel, carboplatin, and toripalimab in patients with ESCC. Twenty patients underwent surgery, and a complete pathologic response was achieved in 11 patients (55%).

In addition to the studies mentioned, four other studies have investigated the role of HFR in patients with esophageal cancer who have undergone non-surgical treatment strategies.

Ma et al. (35) conducted a prospective study comparing CFR (60 Gy in 30 fractions) to a moderately hypofractionated regimen (54-60 Gy in 18-20 fractions). Both groups received concurrent chemotherapy with paclitaxel/cisplatin. The study used a definitive CRT approach. The results showed that while overall survival was similar between the two groups, the rate of locoregional failure was significantly lower in patients treated with HFR (27% vs. 47.3%).

Walterbos et al. (40), Murray et al. (2019) (41), and Borg et al. (2020) (42)conducted studies using a palliative approach, with HFR administered to relieve dysphagia. These studies used regimens of 20 Gy in 5 fractions, 30 Gy in 10 fractions, and 39 Gy in 13 fractions, respectively. The results showed that HFR resulted in dysphagia improvement in over 70% of patients.

Zhou et al. (2023) conducted a study in which 58 patients with unresectable ESCC were treated with hypofractionated CRT (60 Gy in 24 fractions concurrent with S-1). The study achieved a high objective response rate of 91.3%, with 60.3% of patients achieving a complete response according to RECIST criteria (43).

The present study’s findings and previous research consistently demonstrate the effectiveness of hypofractionated CRT for esophageal cancer. This approach offers high rates of pathologic response and dysphagia resolution (**Table 7**).

**Table 7:** Esophageal cancer pathologic response to neoadjuvant HFR in similar studies.

Study	Patient Number	Complete Response	Near Complete Response
**Jiang et al. (39)**	20	11 (55%)	–
**Walsh et al. (19)**	52	13 (25%)	–
**Lyu et al. (18)**	42	14 (33.3%)	–
**This study**	32	17 (53.1%)	6 (18.8%)

In terms of safety, induction chemotherapy followed by hypofractionated CRT has been shown to have a favorable toxicity profile. The most common severe hematologic toxicity observed in the present study was grade 3 neutropenia (15.6%), while the most common severe non-hematologic toxicity was grade 2 or higher odynophagia/esophagitis (37.8%).

Studies by Lyu et al. (18), and Sridharan et al. (44) investigated HFR with RT fraction sizes of 2.33 to 3 Gy in esophageal cancer patients. These studies reported grade 3 esophagitis and grade 3 neutropenia as the most common toxicities, although none led to treatment discontinuation.

There are concerns regarding the potential for acute toxicities such as esophagitis and late toxicities such as fistulas, pneumonitis, cardiac toxicities, or hematologic toxicities associated with HFR for esophageal cancer treatment. However, studies to date have not shown any statistically significant differences in the incidence of grade 3 or higher toxicities between patients treated with CFR regimens (2Gy/fraction) and those treated with HFR regimens (3Gy/fraction) (18, 35).

Our study breaks new ground in Iran by being the first to adopt this specific approach for esophageal cancer treatment. A team of highly regarded experts in Iran conducted the study, which involved a substantial number of patients.

Our study demonstrates a significant step forward in the evaluation of hypofractionated CRT for esophageal cancer in Iran. However, certain limitations need to be addressed in future research. First, the non-randomized, single-arm design of the study introduces the possibility of selection bias, which could potentially affect the interpretation of the results. Second, the majority of patients included in this study had Esophageal ESCC. Therefore, generalizing the study’s findings to patients with EAC may not be fully justified.

## Conclusion

Preoperative induction chemotherapy followed by HFR with concurrent chemotherapy has a low toxicity profile, manageable side effects, good tolerance, and significant efficacy by offering high rates of pathologic response in the treatment of patients with esophageal cancer.

## Data Availability

The original contributions presented in the study are included in the article/supplementary material. Further inquiries can be directed to the corresponding author.

## References

[1] Farhood B Geraily G Alizadeh A. Incidence and mortality of various cancers in Iran and compare to other countries: a review article. Iranian J Public Health. (2018) 47:309. PMC5971166 29845017

[2] DS KOLAH Sajadi A Radmard AR Khademi H. Five common cancers in Iran. Arch Iran Med. (2010) 13:143–6. 20187669

[3] Oppedijk V van der Gaast A van Lanschot JJ van Hagen P van Os R van Rij CM. Patterns of recurrence after surgery alone versus preoperative chemoradiotherapy and surgery in the CROSS trials. J Clin Oncol. (2014) 32:385–91. doi: 10.1200/JCO.2013.51.2186 24419108

[4] Shapiro J Van Lanschot JJB Hulshof MC van Hagen P van Berge Henegouwen MI Wijnhoven BP. Neoadjuvant chemoradiotherapy plus surgery versus surgery alone for oesophageal or junctional cancer (CROSS): long-term results of a randomised controlled trial. Lancet Oncol. (2015) 16:1090–8. doi: 10.1016/S1470-2045(15)00040-6 26254683

[5] Yang H Liu H Chen Y Zhu C Fang W Yu Z. Neoadjuvant chemoradiotherapy followed by surgery versus surgery alone for locally advanced squamous cell carcinoma of the esophagus (NEOCRTEC5010): a phase III multicenter, randomized, open-label clinical trial. J Clin Oncol. (2018) 36:2796. doi: 10.1200/JCO.2018.79.1483 30089078 PMC6145832

[6] Ancona E Ruol A Santi S Merigliano S Chiarion Sileni V Koussis H. Only pathologic complete response to neoadjuvant chemotherapy improves significantly the long term survival of patients with resectable esophageal squamous cell carcinoma: final report of a randomized, controlled trial of preoperative chemotherapy versus surgery alone. Cancer: Interdiscip Int J Am Cancer Soc. (2001) 91:2165–74. doi: 10.1002/(ISSN)1097-0142 11391598

[7] Rohatgi PR Swisher SG Correa AM Wu TT Liao Z Komaki R. Failure patterns correlate with the proportion of residual carcinoma after preoperative chemoradiotherapy for carcinoma of the esophagus. Cancer. (2005) 104:1349–55. doi: 10.1002/cncr.21346 16130133

[8] Schneider PM Baldus SE Metzger R Kocher M Bongartz R Bollschweiler E. Histomorphologic tumor regression and lymph node metastases determine prognosis following neoadjuvant radiochemotherapy for esophageal cancer: implications for response classification. Ann Surg. (2005) 242:684–92. doi: 10.1097/01.sla.0000186170.38348.7b PMC1409844 16244542

[9] Brücher BL Becker K Lordick F Fink U Sarbia M Stein H. The clinical impact of histopathologic response assessment by residual tumor cell quantification in esophageal squamous cell carcinomas. Cancer. (2006) 106:2119–27. doi: 10.1002/cncr.21850 16607651

[10] Langer R Ott K Feith M Lordick F Siewert J-R Becker K. Prognostic significance of histopathological tumor regression after neoadjuvant chemotherapy in esophageal adenocarcinomas. Modern Pathol. (2009) 22:1555–63. doi: 10.1038/modpathol.2009.123 19801967

[11] Meredith KL Weber JM Turaga KK Siegel EM McLoughlin J Hoffe S. Pathologic response after neoadjuvant therapy is the major determinant of survival in patients with esophageal cancer. Ann Surg Oncol. (2010) 17:1159–67. doi: 10.1245/s10434-009-0862-1 20140529

[12] Lorenzen S Thuss-Patience P Al-Batran S Lordick F Haller B Schuster T. Impact of pathologic complete response on disease-free survival in patients with esophagogastric adenocarcinoma receiving preoperative docetaxel-based chemotherapy. Ann Oncol. (2013) 24:2068–73. doi: 10.1093/annonc/mdt141 23592699

[13] Chirieac LR Swisher SG Ajani JA Komaki RR Correa AM Morris JS. Posttherapy pathologic stage predicts survival in patients with esophageal carcinoma receiving preoperative chemoradiation. Cancer. (2005) 103:1347–55. doi: 10.1002/cncr.20916 15719440

[14] Ritter M. Rationale, conduct, and outcome using hypofractionated radiotherapy in prostate cancer. Semin Radiat Oncol. (2008) 18. doi: 10.1016/j.semradonc.2008.04.007 PMC2674313 18725112

[15] Song Y Ma J Hu L Zhou W Chen E Zhang W. Phase I/II study of hypofractioned radiation with three-dimensional conformal radiotherapy for clinical T3-4N0-1M0 stage esophageal carcinoma. Technol Cancer Res Treat. (2011) 10:25–30. doi: 10.7785/tcrt.2012.500176 21214285

[16] Gamez ME Agarwal M Hu KS Lukens JN Harrison LB. Hypofractionated palliative radiotherapy with concurrent radiosensitizing chemotherapy for advanced head and neck cancer using the “QUAD-SHOT regimen. Anticancer Res. (2017) 37:685–91. doi: 10.21873/anticanres 28179317

[17] Shaitelman SF Schlembach PJ Arzu I Ballo M Bloom ES Buchholz D. Acute and short-term toxic effects of conventionally fractionated vs hypofractionated whole-breast irradiation: a randomized clinical trial. JAMA Oncol. (2015) 1:931–41. doi: 10.1001/jamaoncol.2015.2666 PMC4635441 26247543

[18] Lyu J Liu T Li T Li F Wang Q Wang J. Comparison of efficacy, safety, and costs between neoadjuvant hypofractionated radiotherapy and conventionally fractionated radiotherapy for esophageal carcinoma. Cancer Med. (2019) 8:3710–8. doi: 10.1002/cam4.2250 PMC6639169 31119872

[19] Walsh TN Noonan N Hollywood D Kelly A Keeling N Hennessy TP. A comparison of multimodal therapy and surgery for esophageal adenocarcinoma. New Engl J Med. (1996) 335:462–7. doi: 10.1056/NEJM199608153350702 8672151

[20] Kabre RS Kamble K. Feasibility of hypofractionated radiotherapy for locally advanced cancer esophagus. Ann Med Health Sci Res. (2017) 7.

[21] Wang J Yu J Jiang Y Pei D Zhu H Wang J. Hypofractionated radiotherapy in combination with chemotherapy improves outcome of patients with esophageal carcinoma tracheoesophageal groove lymph node metastasis. Front Oncol. (2020) 10:1540. doi: 10.3389/fonc.2020.01540 32984011 PMC7484476

[22] Ajani J Xiao L Roth J Hofstetter W Walsh G Komaki R. A phase II randomized trial of induction chemotherapy versus no induction chemotherapy followed by preoperative chemoradiation in patients with esophageal cancer. Ann Oncol. (2013) 24:2844–9. doi: 10.1093/annonc/mdt339 PMC3937600 23975663

[23] Yoon DH Jang G Kim JH Kim Y-H Kim JY Kim HR. Randomized phase 2 trial of S1 and oxaliplatin-based chemoradiotherapy with or without induction chemotherapy for esophageal cancer. Int J Radiat Oncol Biol Physics. (2015) 91:489–96. doi: 10.1016/j.ijrobp.2014.11.019 25680595

[24] Fang T Oh Y Szabo A Khan A Dua K. Utility of dysphagia grade in predicting endoscopic ultrasound T-stage of non-metastatic esophageal cancer. Dis Esophagus. (2016) 29:642–8. doi: 10.1111/dote.2016.29.issue-6 26382588

[25] Burgart LJ Chopp WV Jain D. Protocol for the examination of specimens from patients with carcinoma of the esophagus. (2021). Available online at: http://www.cap.org/cancerprotocols.

[26] Aledavood SA Sales SS Anvari K Forghani MN Memar B Torghabeh AE. Evaluation of Tumor resectability rate and pathologic response to preoperative concurrent chemoradiotherapy in locally advanced proximal gastric and esophagogastric junction adenocarcinomas: a clinical trial. Int J Cancer Manag. (2017) 10. doi: 10.5812/ijcm

[27] Wu T-T Chirieac LR Abraham SC Krasinskas AM Wang H Rashid A. Excellent interobserver agreement on grading the extent of residual carcinoma after preoperative chemoradiation in esophageal and esophagogastric junction carcinoma: a reliable predictor for patient outcome. Am J Surg Pathol. (2007) 31:58–64. doi: 10.1097/01.pas.0000213312.36306.cc 17197919

[28] Cosset J-M Mornex F Eschwège F. Hypofractionnement en radiothérapie: l’éternel retour. Cancer/Radiothérapie. (2013) 17:355–62. doi: 10.1016/j.canrad.2013.06.027 23969245

[29] Jiang W Wang JY Wang JB Liang J Hui ZG Wang XZ. Hypofractionated radiotherapy for medically inoperable stage I non-small cell lung cancer. Thorac Canc. (2016) 7:296–303. doi: 10.1111/1759-7714.12327 PMC4846617 27148414

[30] Urbanic JJ Wang X Bogart JA Stinchcombe TE Hodgson L Schild SE. Phase 1 study of accelerated hypofractionated radiation therapy with concurrent chemotherapy for stage III non-small cell lung cancer: CALGB 31102 (Alliance). Int J Radiat Oncol Biol Physics. (2018) 101:177–85. doi: 10.1016/j.ijrobp.2018.01.046 PMC6173195 29487024

[31] Benjamin LC Tree AC Dearnaley DP. The role of hypofractionated radiotherapy in prostate cancer. Curr Oncol Rep. (2017) 19:1–9. doi: 10.1007/s11912-017-0584-7 28343352 PMC5366169

[32] Dearnaley D Syndikus I Mossop H Khoo V Birtle A Bloomfield D. Conventional versus hypofractionated high-dose intensity-modulated radiotherapy for prostate cancer: 5-year outcomes of the randomised, non-inferiority, phase 3 CHHiP trial. Lancet Oncol. (2016) 17:1047–60. doi: 10.1016/S1470-2045(16)30102-4 PMC4961874 27339115

[33] Lalani N Paszat L Sutradhar R Thiruchelvam D Nofech-Mozes S Hanna W. Long-term outcomes of hypofractionation versus conventional radiation therapy after breast-conserving surgery for ductal carcinoma in *situ* of the breast. Int J Radiat Oncol Biol Physics. (2014) 90:1017–24. doi: 10.1016/j.ijrobp.2014.07.026 25220719

[34] Finnegan TS Bhatt NH Shaughnessy JN Perez C Redman R Silverman C. Cyclical hypofractionated radiotherapy technique for palliative treatment of locally advanced head and neck cancer: institutional experience and review of palliative regimens. J Community Support Oncol. (2016) 14:29–36. doi: 10.12788/jcso.0201 26870840

[35] Ma JB Wei L Chen EC Qin G Song YP Chen XM. Moderately hypofractionated conformal radiation treatment of thoracic esophageal carcinoma. Asian Pac J Cancer Prev. (2012) 13:4163–7. doi: 10.7314/APJCP.2012.13.8.4163 23098423

[36] Ma B Gao P Song Y Huang X Wang H Xu Q. Short-course radiotherapy in neoadjuvant treatment for rectal cancer: a systematic review and meta-analysis. Clin colorectal Canc. (2018) 17:320–30. e5. doi: 10.1016/j.clcc.2018.07.014 30243484

[37] Ngan SY Burmeister B Fisher RJ Solomon M Goldstein D Joseph D. Randomized trial of short-course radiotherapy versus long-course chemoradiation comparing rates of local recurrence in patients with T3 rectal cancer: Trans-Tasman Radiation Oncology Group trial 01. 04. J Clin Oncol. (2012) 30:3827–33. doi: 10.1200/JCO.2012.42.9597 23008301

[38] Latkauskas T Pauzas H Kairevice L Petrauskas A Saladzinskas Z Janciauskiene R. Preoperative conventional chemoradiotherapy versus short-course radiotherapy with delayed surgery for rectal cancer: results of a randomized controlled trial. BMC Canc. (2016) 16:1–7. doi: 10.1186/s12885-016-2959-9 PMC5131536 27903247

[39] Jiang N Zhang J Guo Z Wu Y Zhao L Kong C. Short-course neoadjuvant radiotherapy combined with chemotherapy and toripalimab for locally advanced esophageal squamous cell carcinoma (SCALE-1): a single-arm phase Ib clinical trial. J Immunother Cancer. (2024) 12. doi: 10.1136/jitc-2023-008229 PMC10806572 38199609

[40] Walterbos NR Fiocco M Neelis KJ van der Linden YM Langers AM Slingerland M. Effectiveness of several external beam radiotherapy schedules for palliation of esophageal cancer. Clin Trans Radiat Oncol. (2019) 17:24–31. doi: 10.1016/j.ctro.2019.04.017 PMC6517531 31193091

[41] Murray LJ Din OS Kumar VS Dixon LM Wadsley JC. Palliative radiotherapy in patients with esophageal carcinoma: a retrospective review. Pract Radiat Oncol. (2012) 2:257–64. doi: 10.1016/j.prro.2011.12.002 24674161

[42] Borg D Sundberg J Brun E Kjellén E Petersson K Hermansson M. Palliative short-course hypofractionated radiotherapy followed by chemotherapy in esophageal adenocarcinoma: the phase II PALAESTRA trial. Acta Oncol. (2020) 59:212–8. doi: 10.1080/0284186X.2019.1670861 31564184

[43] Zhou R Luo G Guo S Wu Y Luo Q Wang D. Moderately hypo-fractionated radiotherapy combined with S-1 in inoperable locally advanced esophageal squamous cell carcinoma: A prospective, single-arm phase II study (GASTO-1045). Front Oncol. (2023) 13:1138304. doi: 10.3389/fonc.2023.1138304 36969023 PMC10036360

[44] Sridharan S Day F Loh J Lynam J Smart J Holt B. Phase I trial of hypofractionated chemoradiotherapy in the palliative management of esophageal and gastro-esophageal cancer. Radiat Oncol. (2022) 17:158. doi: 10.1186/s13014-022-02127-x 36104707 PMC9472395

